# Exploring Obesity Subtypes: Cluster Analysis of Eating Behaviors, Food Addiction, and Psychopathology in Turkish Adults Seeking Obesity Treatment

**DOI:** 10.3390/nu17243823

**Published:** 2025-12-06

**Authors:** Orçun Ortaköylü, Ayşe Gökçen Gündoğmuş, Sibel Örsel

**Affiliations:** 1 Department of Psychiatry, Malatya Training and Research Hospital, 44090 Malatya, Turkey; 2 Department of Mental Health, General Directorate of Public Health, Republic of Turkey Ministry of Health, 06800 Ankara, Turkey; gokcengonen@gmail.com; 3 Department of Psychiatry, Ankara Etlik City Hospital, University of Health Sciences, 06170 Ankara, Turkey

**Keywords:** obesity, food addiction, cluster analysis, problematic eating attitudes, obesity subtypes, maladaptive eating

## Abstract

**Background/Objectives**: Obesity results from the complex interplay of biological, psychological, and sociocultural factors. Psychiatric comorbidities and maladaptive eating attitudes are possible contributors to this complexity. Despite accumulating research, current treatment approaches often fail to achieve lasting success, possibly due to the tendency to approach obesity as a uniform condition despite its heterogeneity. This study aimed to identify distinct subgroups of adults with obesity based on their eating behaviors, psychopathology, and food addiction (FA). **Methods**: The study included 202 adults seeking obesity treatment at Ankara Dışkapı Training and Research Hospital, Turkey. Participants completed the Three Factor Eating Questionnaire-R18, Yale Food Addiction Scale, Night Eating Questionnaire, and Symptom Checklist-90-R (SCL-90-R). Psychiatric diagnoses were confirmed through clinician-administered semi-structured interviews (SCID-5-CV). Two-step cluster analysis was performed using z-standardized variables of eating and psychopathology measures. Subsequent comparisons were conducted between the identified subgroups. **Results**: Two distinct clusters were identified and described in terms of adaptive and maladaptive eating traits: an Adaptive group (*n* = 92, 45.5%) and a Maladaptive group (*n* = 110, 54.5%). The Maladaptive cluster exhibited higher levels of emotional eating, uncontrolled eating, night eating, FA, and general psychopathology (all *p* < 0.001), along with a greater prevalence of major depressive disorder and binge eating disorder. **Conclusions**: Our findings indicate that psychopathology and maladaptive eating behaviors meaningfully distinguish two obesity phenotypes. Nearly half of individuals with obesity, characterized by maladaptive eating traits, FA, or elevated psychopathology, may constitute a subgroup that would particularly benefit from psychoeducation or targeted psychiatric intervention.

## 1. Introduction

Obesity is an increasingly prevalent global public health concern characterized by complex interactions among biological, psychological, and sociocultural factors. Despite the availability of diverse behavioral, pharmacological, and surgical interventions, long-term weight loss success remains limited [1,2]. One potential reason for these suboptimal outcomes is the absence of a more individualized approach, even though various factors have been linked with obesity [3]. An increasing number of studies have shown that obesity frequently coexists with psychiatric co-morbidities including depression, anxiety disorders, binge eating disorder (BED), bulimia nervosa (BN) along with maladaptive eating attitudes such as emotional eating (EE), night eating (NE), cognitive restraint (CR), uncontrolled eating (UE), and food addiction (FA) [4,5,6,7]. BED is the eating disorder most frequently associated with obesity, although prevalence estimates vary widely across studies, ranging from 2% to 49% [8]. The concept of food addiction (FA), adapted from substance addiction, has attracted increasing attention due to its correlation with other eating disorders, obesity, and its diagnostic validity [9]. Studies show a significantly high prevalence of FA among bariatric surgery candidates, and some studies report poorer outcomes with these patients [10,11]. Shared features with substance addiction, including cravings, loss of control, and continuation despite consequences, along with accumulating neuroscientific findings, reinforce the validity of food addiction [12,13]. Taken together, the aforementioned studies are in line with a narrative review by Nightingale and Cassin (2019), which synthesized findings on multiple forms of disordered eating including BED, BN, EE and FA among individuals with excess weight/obesity, reported that 18.6% to 48.3% of participants met diagnostic criteria for BED and highlighted that binge eating, loss-of-control eating, FA, and EE share many common correlates, thereby underlining the substantial overlap between obesity and disordered eating and underscoring the need to identify subgroups characterized by distinct psychological and behavioral profiles [14].

With the expanding body of research highlighting the psychiatric and psychological aspects of obesity and their relevance to treatment outcomes, psychiatry has assumed an increasingly important role in obesity management and has become a component of bariatric surgery programs [15]. Troisi recently introduced the concept of bariatric psychiatry as a new subspecialty, underscoring the field’s growing importance within obesity medicine [15].

Building on this background, we investigated adult patients seeking obesity treatment at the Obesity Center of a state hospital in Ankara, Turkey, focusing on psychopathology, disordered eating behaviors, and food addiction. In the present study, we use “maladaptive eating” as a descriptive, non-diagnostic umbrella construct summarizing a range of problematic and disordered eating behaviors, including EE, NE, FA, bingeing, and UE, in line with previous studies that have used a similar umbrella concept to describe such eating behaviors [16,17,18].

Using two-step cluster analysis, we aimed to identify and compare subgroups, particularly those exhibiting greater psychopathology and maladaptive eating, who may benefit more from psychiatric evaluation. In addition to many similar studies that rely on self-report scales for eating disorders and psychiatric diagnoses, in our study, we utilized both self-report scales and structured clinical assessments for psychiatric diagnoses.

We hypothesized that increased level of psychopathology and maladaptive eating behaviors would characterize a distinct obesity phenotype, and that comparisons between subgroups based on clinical data would further support this distinction.

## 2. Materials and Methods

### 2.1. Subjects

Our study included a total of 205 adult volunteers who applied to the Obesity Center of Ankara Dışkapı Training and Research Hospital (Ankara, Turkey) between March 2019 and March 2020, primarily seeking treatment for obesity. The workflow of our center involved accepting all individuals requesting obesity treatment. For candidates of surgical intervention, psychiatric evaluation was a mandatory component of the preoperative approval process. These patients were informed about the study, and written consent was obtained for the use of their clinical data in research. Patients who initially applied for medical (non-surgical) treatment were also informed about the study and provided consent for their data to be included; if they later opted for surgical treatment, their data were incorporated into the pre-surgery assessment files. Of the 205 participants, 124 expressed a preference for surgical treatment and 81 for medical treatment. The sample consisted of 163 females and 39 males. Three participants were excluded due to incomplete data, yielding a final study sample of 202 individuals (163 females, 39 males). The Inclusion criteria were having a body mass index (BMI) of ≥30 kg/m^2^, being between 18 and 65 years of age; and providing informed consent to participate in the research and the exclusion criteria were having active alcohol or drug use during the assessment; the presence of active psychosis, mania, suicide risk, or dementia; having a physical or mental condition that would prevent completion of the study forms; and having a body mass index (BMI) below 30 kg/m^2^. During recruitment, conditions known to directly contribute to obesity, such as Cushing’s syndrome, acute chronic corticosteroid use, genetically defined syndromes causing obesity (Prader-Willi, etc.), or untreated hypothyroidism, were reviewed; however, no such cases were identified. Because obesity and Type-2 Diabetes Mellitus are strongly intertwined [19], individuals with Type-2 Diabetes Mellitus were not excluded. This approach was chosen to avoid selection bias and to better reflect real-world populations presenting to clinical care. Ethical approval for the present study was obtained from the local hospital’s ethical committee.

### 2.2. Assessment and Measures

Data, such as age, sex, height, weight, educational status, occupation, and marital status of the participants, were obtained through the sociodemographic data form as self-reports prior to clinical assessment. BMI values were calculated by a clinician using self-reported height and weight. Diagnostic assessment of participants was made by trained psychiatrists through the semi-structured clinical interview for the DSM-5 (SCID-5-CV) [20]. Validated Turkish versions of the following scales were applied to assess eating patterns and psychopathology.

#### 2.2.1. Three-Factor Eating Questionnaire (TFEQ-R18)

TFEQ-R18 was first developed by Stunkard and Messick (1985) to measure the behavioral and cognitive components of eating, later revised by Karlsson et al. [21], resulting in a form containing three factors: Uncontrolled eating, cognitive restraint, and emotional eating [22]. Cronbach’s alpha value for Turkish version was 0.71 [23]. Responses to each of the 18 items are rated on a 4-point scale (1–4), and item scores are summed separately to obtain raw subscale scores for CR, UE, and EE, respectively. The raw scale scores are transformed to a 0–100 scale [((raw score−lowest possible raw score)/possible raw score range) × 100] [21]. Higher scores from the subscales are interpreted that the subscale eating behavior in the individual is high in comparison, with no specified cut-off values.

#### 2.2.2. Night Eating Questionnaire (NEQ)

NEQ is a 14-item, 5-point Likert-type instrument developed by Allison et al. (2008) [24] used to assess the symptoms defined for night eating syndrome (NES). The questionnaire measures four aspects, including nocturnal feeding, evening hyperphagia, morning anorexia, and sleep. The night eating syndrome was first described by Stunkard et al. as characterized by morning anorexia, evening hyperphagia, and insomnia [24]. The Cronbach alpha was reported to be 0.69 for the Turkish version of the questionnaire [25].

#### 2.2.3. Yale Food Addiction Scale (YFAS)

The YFAS was developed by Gearhardt et al. (2009), adapting the DSM-IV-TR substance dependence diagnostic criteria for use in assessing addictive-like eating behaviors related to highly processed food consumption [26]. The Turkish validity and reliability study was conducted by Sevinçer et al. (2015) among bariatric surgery candidates, demonstrating good internal consistency (Cronbach’s α = 0.859) [27]. The scale consists of 26 items, 25 of which are rated on a Likert-type scale, while the 26th item assesses food choices and is not included in scoring. The items correspond to seven diagnostic criteria for substance dependence, with an additional subscale assessing clinically significant distress. The items of the scale resemble symptoms of substance use disorders as stated in the DSM-IV-TR. Two scoring approaches are recommended by the developers: symptom count and diagnostic scoring. The symptom count method represents the total number of symptom criteria met (ranging from 0 to 7), whereas the diagnostic scoring yields a dichotomous outcome indicating the presence or absence of food addiction. According to the diagnostic criteria, food addiction is identified when three or more of the seven symptom criteria are met, accompanied by clinically significant impairment and/or distress [7].

#### 2.2.4. Symptom Check List (SCL-90-R)

The Symptom Checklist-90-Revised (SCL-90-R) was developed by Derogatis to assess the level of psychological distress, negative stress responses, and a broad range of psychiatric symptoms experienced by individuals [28]. It is a 90-item, 5-point Likert-type self-report scale, with item scores ranging from 0 to 4. The instrument has nine subscales: Somatization, Obsessive–Compulsive, Interpersonal Sensitivity, Depression, Anxiety, Hostility, Phobic Anxiety, Paranoid Ideation, and Psychoticism, and additional items. Three global calculations were defined, namely Global Severity Index (GSI), Positive Symptom Distress Index (PSDI), and Positive Symptom Total (PST) provide overall psychological distress and level of psychiatric symptomatology. The Turkish adaptation of the scale was validated by Dağ (1991), demonstrating high reliability and construct validity for use [29].

### 2.3. Statistical Analysis

The data were analyzed with SPSS 25 for Windows. Continuous data were expressed as mean ± standard deviation (SD), and categorical variables as frequencies and percentages.

Clusters among the 202 participants were evaluated using a two-step cluster analysis. The two-step cluster analysis is chosen because it does not require a priori specification of the number of clusters, can automatically select the best-fitting number of clusters, and accommodates multiple continuous variables. To minimize bias from differing measurement scales, all variables were standardized to z-scores before performing the cluster analysis. The log-likelihood distance measure was calculated. Goodness-of-fit was assessed using both the Bayesian Information Criterion (BIC) and the average silhouette coefficient. Lower BIC values indicate superior model fit [30], whereas higher silhouette values reflect greater within-cluster cohesion and between-cluster separation. Silhouette coefficient values > 0.5 were considered good, 0.2–0.5 fair, and <0.2 poor [31]. Criteria for selecting the final number of clusters and distribution model included adequate goodness-of-fit and clinical interpretability [32]. The primary indicators used in the cluster analysis were selected on the basis of previous research focusing on subtyping obesity in regard to various maladaptive eating patterns, psychopathology, and eating disorders [9,33,34,35,36,37], along with clinical relevance. In line with our hypothesis, continuous psychometric variables regarding problematic eating behaviors and general psychiatric symptoms were preferred, as continuous variables preserve the distribution and allow more sensitive detection of phenotypes in cluster analysis. Thus, for the analysis, continuous variables related to problematic eating behaviors (EE, UE, and EE subscale scores of TFEQ, total score of NEQ, symptom count of YFAS), and overall psychological distress (Global Severity Index of SCL-90R) were chosen. Age, BMI, and weight were initially selected as variables but showed almost no predictive importance and were excluded to obtain a more stable model.

After determining the clusters, differences were explored using chi-square tests (χ^2^) for categorical variables (categorical sociodemographic and clinical assessment data), and effect sizes were expressed as Cramer’s V. Independent samples *t*-tests were employed to compare continuous variables (scale scores, age, BMI, height, weight), and effect sizes were reported as Cohen’s d. For descriptive visualization, a radar chart was constructed to illustrate the relative profiles of the two clusters based on the variables used for clustering. BMI and age were added to the figure to demonstrate that the clusters were similar in these regards. Prior to plotting, all variables were z-standardized to ensure comparability across scales.

## 3. Results

### 3.1. Characteristics and Scale Scores of the Study Sample

The study population consisted of 163 females (80.7%) and 39 males (19.3%) participants with a mean age of 40.04 ± 11.30 years. Most participants had Upper Secondary Education (33.7%), followed by Higher Education (28.7%). Twenty-nine participants (19.9%) were diagnosed with MDD, and 38 (18.8%) with BED. The mean subscale scores of the TFEQ were 44.71 ± 25.06 for UE, 52.09 ± 33.24 for EE, and 52.28 ± 19.75 for CR. The mean NEQ score was 17.77 ± 9.43. The number of food addiction criteria met by participants was 4.26 ± 1.75, and finally, 48.0% (*n* = 97) of participants met the threshold for FA in dichotomous assessment (Table 1).

### 3.2. Cluster Analysis Results

The best cluster solution was the two-cluster model, which was also selected automatically by a two-step cluster procedure. This solution showed a low BIC value of 749.215 and an average silhouette coefficient of 0.50, indicating good cluster quality. The highest ratio of distance measures for the log-likelihood criterion (2.872), suggesting clear separation between the clusters, was highest for the two-cluster model. Although the three-cluster solution showed a slightly lower (more favorable) BIC (737.964), its silhouette coefficient (0.30) and lower ratio of distance measures (1.723) indicated weaker separation between clusters. Therefore, the two-cluster model was retained as the optimal and most interpretable solution.

Out of 202 participants, 92 patients (45.5%) went under Cluster 1 and 110 (54.5%) under Cluster 2. The indicator variables with the highest contribution to the clustering were the EE subscale of TFEQ with the value of 1.0, followed by UE (0.67), and YFAS symptom score (0.43). The latter-excluded variables, BMI, weight, and age, showed low predictor importance values of 0.07, 0.05, and 0.03, respectively. As shown in Table 2 and Table 3, Cluster 2 exhibited higher levels of maladaptive eating patterns EE, UE, NE, FA, and overall psychopathology, whereas Cluster 1 demonstrated higher CR and lower psychopathological symptoms. Because EE, FA, and UE were the strongest contributors to the cluster solution, and as “maladaptive eating” is used in the obesity and eating-behavior literature as a concise umbrella construct encompassing these behaviors [16,17], we considered it inclusive and relatively non-stigmatizing label; although it does not fully capture every nuance of the construct, it remains coherent with our statistical findings. The clusters were therefore labeled “Adaptive” (Cluster 1) and “Maladaptive” (Cluster 2).

#### 3.2.1. Inter-Cluster Comparison of Sociodemographic Properties, Food Addiction, and Clinical Data

No significant differences were observed between the clusters in terms of sex, education level, or occupation (*p* > 0.05). However, the prevalence of MDD, BED, and FA was significantly higher in the Maladaptive cluster (*p* < 0.05).

#### 3.2.2. Comparison of Groups in Terms of Scale Scores and Sociodemographic Numerical Data

There were no significant differences between the clusters in terms of age or BMI. Scores for TFEQ-EE, TFEQ-UE, NEQ, and FA symptoms were higher in the “Maladaptive” group, whereas the “Adaptive” group demonstrated higher TFEQ-CR scores (Table 3). Psychopathology scores assessed with the SCL-90-R were significantly higher in the “Maladaptive” group compared to the “Adaptive” group (*p* < 0.001). Cohen’s d values were calculated to quantify the magnitude of group differences. Effect sizes for psychological and eating-behavior variables were consistently large to very large. Specifically, d values ranged from 0.84 to 2.47 across the YFAS criteria, TFEQ subscales, and SCL-90-R symptom dimensions, reflecting clinically meaningful differences between groups. The strongest effects were observed for EE (d = 2.47) and UE (d = 1.83) subscales of TFEQ, while other measures, such as the Global Symptom Index (d = 1.32), Depression (d = 1.20), and Anxiety (d = 1.14), also indicated large effects.

**Table 3:** Comparison of Scale Scores, Age, and BMI between Clusters.

	Cluster 1 (Adaptive)		Cluster 2 (Maladaptive)		F	*p*	*d*
	Mean	SD	Mean	SD			
Age (years)	39.86	11.65	40.21	11.06	0.048	0.827	0.03
BMI (kg/m^2^)	42.73	5.57	43.30	5.35	0.550	0.459	0.10
Height (m)	1.64	0.08	1.63	0.10	1.137	0.288	0.15
Weight (kg)	115.36	19.84	114.75	18.19	0.051	0.822	0.03
YFAS symptom score (0–7)	3.16	1.50	5.15	1.41	93.261	<0.001 *	**1.36**
TFEQ-R18: UE	26.25	16.43	60.17	20.11	167.965	<0.001 *	**1.83**
TFEQ-R18: EE	23.91	21.72	75.66	20.33	304.826	<0.001 *	**2.47**
TFEQ-R18: CR	61.11	19.54	44.90	16.73	40.358	<0.001 *	0.90
NEQ: Total Score	12.42	5.85	22.40	9.54	76.561	<0.001 *	**1.24**
SCL90-R: GSI	0.61	0.40	1.33	0.64	87.594	<0.001 *	**1.32**
SCL90-R: Somatization	0.88	0.68	1.61	0.88	42.351	<0.001 *	0.92
SCL90-R: Obsessive Compulsive	0.80	0.54	1.64	0.74	82.61	<0.001 *	**1.29**
SCL90-R: Interpersonal Sensitivity	0.81	0.66	1.66	0.95	53.4	<0.001 *	1.03
SCL90-R: Depressive	0.70	0.53	1.55	0.83	72.461	<0.001 *	**1.20**
SCL90-R: Anxiety	0.40	0.34	1.05	0.70	65.363	<0.001 *	1.14
SCL90-R: Hostility	0.48	0.46	1.07	0.76	42.444	<0.001 *	0.92
SCL90-R: Phobic Anxiety	0.21	0.32	0.71	0.74	35.784	<0.001 *	0.84
SCL90-R: Paranoia	0.55	0.49	1.27	0.79	59.279	<0.001 *	1.09
SCL90-R: Psychoticism	0.30	0.38	0.79	0.61	44.905	<0.001 *	0.95
SCL90-R: PST	32.51	18.63	54.34	18.79	68.112	<0.001 *	1.17

* *p* < 0.05; *p* = significance level; *d*: Cohen’s d values represent standardized mean differences between groups, interpreted as small (0.20–0.49), medium (0.50–0.79), large (0.80–1.19), very large (1.20–1.99), and extremely large (≥2.00) effects; Written in bold: Cohen’s d values higher than 1.19; SD: Standard Deviation; BMI: Body Mass Index; YFAS: Yale Food Addiction Scale; TFEQ: Three-Factor Eating Questionnaire; UE: Uncontrolled Eating; EE: Emotional Eating, CR: Cognitive Restraint; NEQ: Night Eating Questionnaire; SCL90-R: Symptom Check List 90 Revised; GSI: Global Severity Index; PST: Positive Symptom Total.

**Figure 1:**
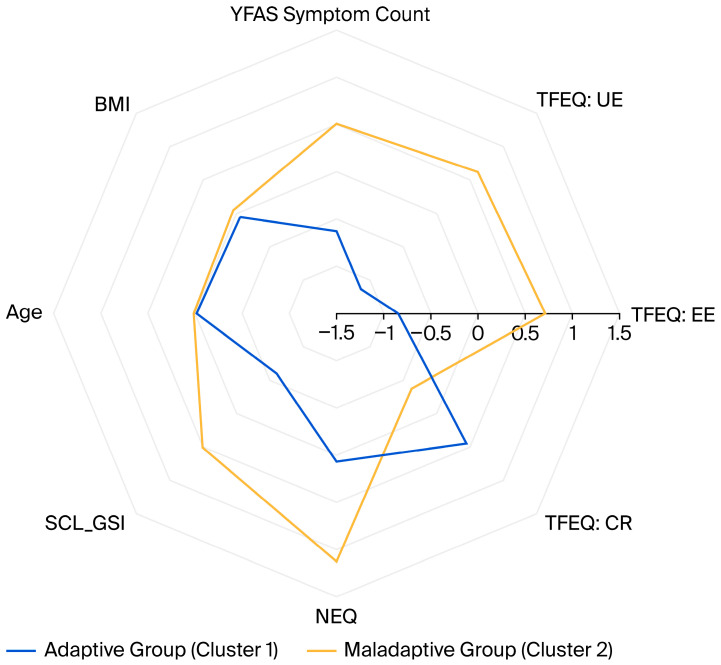
Radar chart comparing the z-standardized mean scores between the groups. YFAS: Yale Food Addiction Scale; BMI: Body mass index; TFEQ: Three-Factor Eating Questionnaire; UE: Uncontrolled Eating; EE: Emotional Eating, CR: Cognitive Restraint; SCL_GSI: Global Severity Index of the SymptomChecklist-90-Revised; NEQ: Night Eating Questionnaire.

For visualization and comparison, Figure 1 presents a radar chart illustrating the two clusters across the main clustering variables, along with age and BMI. Z-standardized scores were plotted to enhance interpretability.

## 4. Discussion

This study aimed to identify and compare subgroups among adults seeking obesity treatment based on psychopathology and disordered eating profiles. Using two-step cluster analysis, two distinct groups emerged that differed markedly in emotional and uncontrolled eating, food addiction, and overall psychological distress despite having similar BMI and sociodemographic characteristics. Because maladaptive eating patterns were the strongest contributors to the cluster solution, and this distinction is consistent with prior literature and our statistical findings, we labeled the clusters “Adaptive” and “Maladaptive”. Comparisons showed that the maladaptive cluster exhibited higher scores with large effect sizes for FA symptoms, NE, UE, depression, obsessive–compulsive symptoms, and general psychological distress, as well as an extremely large effect size for emotional eating, indicating potential clinical relevance. Cognitive restraint was the only eating-related dimension elevated in the Adaptive cluster, and MDD and BED diagnoses were more prevalent in the maladaptive cluster.

Our findings are consistent with earlier studies demonstrating that subgroups characterized by maladaptive eating patterns co-occur with elevated psychiatric symptomatology, suggesting a distinct obesity phenotype [3,9,34,35,36,37,38,39,40,41]. For example, Caroleo et al. (2018) described a cluster of individuals with obesity who showed more frequent BED, anxiety disorders, depression, emotional eating, bingeing, night eating, carbohydrate craving, and snacking [35]. Müller et al. (2014) similarly identified an “emotionally dysregulated” phenotype marked by greater maladaptive eating-disorder symptoms, higher rates of BED, and more severe depression scores [40]. A clustering study by Leombruni et al. (2014) reported a subgroup characterized by higher levels of impulsive eating, depression, poorer quality of life, and more negative body image [36]. More recently, Carbone et al. (2024) identified three clusters, in which a higher frequency of altered eating behaviors was associated with more severe general and eating-related psychopathology, while clusters with a lower frequency of altered eating behaviors exhibited less symptomatology [34]. In line with these findings, our “maladaptive” subgroup displayed substantially elevated emotional and uncontrolled eating, higher rates of binge-eating disorder and major depression, and markedly higher SCL-90-R scores, whereas the “adaptive” subgroup showed comparatively regulated eating behavior and lower psychiatric burden. The pattern parallels phenotypes reported across the abovementioned studies, reinforcing the replicability of such obesity subtypes.

Cognitive restraint is considered a potential precipitating factor for subsequent binge eating episodes [42]. Although the coexistence of CR with eating disorders is generally acknowledged, studies examining the relationships among dietary restraint, obesity, and eating disorders have resulted in differing conclusions. For example, a 2025 study involving 400 participants reported that higher CR scores were associated with lower BMI [43]. In another study, Morin and colleagues experimentally induced cognitive dietary restraint and found no adverse impact on food cravings or body weight compared with a control group [44]. In a longitudinal study, Roberts et al. (2007) [45] reported that, following a 12-week period of chronic stress, individuals who gained weight were those with the highest initial BMI and dietary restraint scores. Participants who maintained their level of dietary restraint during the stress period either maintained or lost weight, and those who significantly reduced their cognitive restraint exhibited weight gain and low mood [45]. In our study, CR scores were significantly lower in the “maladaptive” group. Likewise, Müller et al. reported that their “emotionally dysregulated” cluster, conceptually similar to our “maladaptive” subgroup, did not exhibit meaningfully higher restraint scores (*p* > 0.05) [40]. Similarly, in the study by Aloi et al., which compared clinical profiles in individuals with obesity with and without BED, dietary restraint was described as “not a significant differentiating factor,” showing no strong association with FA; mechanisms “other than the restraint” subscale were considered to be of greater relevance in their analysis [46].

Along with aforementioned maladaptive eating patterns, food addiction, with an expanding body of literature and ongoing debates about its diagnostic validity [47,48], deserves particular consideration. In our study, almost 71% of individuals in the “maladaptive” group met the criteria for FA, indicating a significant co-occurrence between FA and the maladaptive eating/psychopathology profile. This aligns with findings from a nationwide Danish study by Horsager et al. (2021), which emphasized that FA is highly prevalent among individuals with mental disorders, especially those with eating disorders, personality disorders, and mood disorders [37]. Current research has also emphasized shared clinical and neurocognitive features between FA and BED constructs, including dysregulated reward processing, cue-induced craving, emotional dysregulation, impulsivity, and executive dysfunction [49]. Both existing literature and our findings indicate that FA, BED, and other maladaptive eating patterns commonly co-occur, suggesting a possible obesity subtype [50].

In this context, along with the results of this study, both FA and EE may serve as screening tools to identify individuals belonging to the “maladaptive” cluster, for whom stress management could represent a therapeutic focus in obesity treatment, or FA can be an essential treatment target to reduce obesity in these populations [37]. Indeed, randomized controlled trials have demonstrated greater BMI reduction among participants receiving stress management education [51]. Clinical implications emerging from the current literature, together with our findings, highlight that individuals who exhibit greater vulnerability to emotional eating, as seen in the ‘maladaptive’ group, may respond particularly well to interventions aimed at strengthening emotion-regulation skills, including psychotherapeutic approaches specifically designed to reduce emotion dysregulation [52]. Specific nutritional interventions have been proposed based on growing evidence that diet and nutrition influence mental health outcomes. Adopting a healthy dietary pattern, such as the Mediterranean diet, has been associated with reductions in the risk of mood disorders [53]. In this context, the emerging field of nutritional psychiatry [54] may also contribute to designing dietary approaches that support mood regulation while simultaneously guiding structured energy restriction for individuals within the ‘maladaptive’ subtype. In contrast, for individuals in the adaptive group, extensive psychiatric or psychological assessment may be unnecessary and could reduce treatment efficiency by diverting time and motivation away from lifestyle focused or behavioral interventions. In terms of CR, conventional weight-loss approaches emphasize dietary restraint as a means of deliberately restricting food intake to achieve weight loss. Our findings, if replicated in future research, may help generate hypotheses about which individuals might benefit from CR-focused interventions and for whom such approaches may be less applicable hypothetically, individuals in the “adaptive” cluster, who already demonstrate higher CR levels, may not require or may be less responsive to such interventions.

In summary, growing evidence shows that classifying obesity solely by BMI is inadequate for understanding its etiology or guiding individualized treatment strategies [39]. Studies aiming to identify obesity subtypes have highlighted the value of psychological and behavioral profiling in tailoring more effective interventions [33,38]. Our study results support that eating patterns and psychopathology can be used to differentiate phenotypes of obesity through statistical analysis, similar to findings reported in previous studies [36,55]. Based on our findings and previous research, certain obesity subtypes should be evaluated within a psychiatric framework. Primary healthcare providers and dietitians should be educated to recognize these subtypes and to refer affected individuals for psychological assessment rather than focusing solely on increasing physical activity or prescribing restrictive diets.

This study contributes novel data from a Turkish population. Because most prior research in this field has been conducted in Western populations, our findings help broaden the evidence base and support a more global understanding of psychological and eating behavior patterns and subtypes in obesity. A notable strength of the present study is that psychiatric diagnoses were confirmed through clinician-administered SCID-5-CV interviews rather than relying solely on self-report measures. Nonetheless, certain limitations should be acknowledged. First, given the cross-sectional nature of the study, the observed associations cannot establish causal pathways between psychopathology and maladaptive eating behaviors. Second, the sample size is rather small for generalizability on a global scale. Third, our sample was predominantly female, which may restrict the applicability of these profiles to the underrepresented gender. Although this female predominance is partially consistent with national patterns, according to the WHO Europe 2022 report, obesity prevalence in Turkey is higher among women (39.2%) than men (24.4%) [56]; the proportion of women in our sample (approximately 80%) is still higher than expected based on population-level data. Fourth, the study was conducted in a single obesity center, which may limit external validity and restrict generalizability to other settings and health systems. Fifth, we did not collect hormonal or other biological markers, so we were unable to examine whether the behavioral and psychological profiles we identified correspond to distinct biological phenotypes.

Future research should aim to replicate and extend these findings in larger, more diverse, and gender-balanced samples, ideally with inclusion of biological, metabolic, and genetic markers. Longitudinal studies are required to clarify causal relationships between psychopathology, eating behaviors, and obesity phenotypes. Incorporating biological and psychological and eating behavior measures, along with stress-related parameters, could further elucidate the mechanisms linking maladaptive eating and emotional regulation in obesity. Moreover, interventional studies should explore whether incorporating psychoeducation, stress management, and cognitive behavioral or emotion-focused therapies alongside nutritional counseling enhances treatment outcomes. Expanding clustering research across different cultural and socioeconomic settings would improve the generalizability of obesity subtyping. Ultimately, a better understanding of these multidimensional phenotypes may facilitate the development of integrative, obesity care models that include psychoeducation for both patients and healthcare providers, supporting more tailored and effective treatment outcomes.

## 5. Conclusions

In conclusion, our results advance the conceptualization of obesity beyond weight-centric models, emphasizing the role of psychological and eating behavioral variables. The findings obtained in this study suggest that assessing maladaptive eating behaviors and psychopathology could help plan more tailored treatments for obesity, and psychiatric consultation can guide the customization of obesity treatment or play an important role in the treatment of certain phenotypes. From a clinical standpoint, our results suggest that patients in the “Maladaptive” cluster might benefit from earlier and more intensive psychiatric, psychopharmacologic, or cognitive intervention or psychoeducation.

## Figures and Tables

**Table 1:** Distribution of sociodemographic and clinical characteristics of the participants.

		*n* (%)
BMI (kg/m^2)^ (mean ± SD)		43.03 ± 5.44
Age (years) (mean ± SD)		40.04 ± 11.30
Sex	Female	163 (80.7)
	Male	39 (19.3)
Education	Primary Education	36 (17.8)
	Lower Secondary Education	40 (19.8)
	Upper Secondary Education	68 (33.7)
	Higher Education	58 (28.7)
Diagnoses (DSM-5)	Major Depressive Disorder	29 (14.4)
	Anxiety Disorder	20 (9.9)
	Obsessive–Compulsive Disorder	5 (2.5)
	Attention Deficit Hyperactivity Disorder	2 (1)
	Adjustment Disorder	9 (4.5)
	Panic Disorder	2 (1)
Eating and Feeding Disorders (DSM-5)	Pica	2 (1)
	Avoidant/Restrictive Food Intake Disorder	6 (3)
	Bulimia Nervosa	3 (1.5)
	Binge-Eating Disorder	38 (18.8)
Food Addiction		97 (48)

Note: BMI: Body Mass Index; SD: Standard Deviation; DSM-5: Diagnostics and Statistical Manual of Mental Disorders, 5th Edition.

**Table 2:** Comparison of clusters in terms of sociodemographic data, psychiatric diagnosis, and food addiction.

		Cluster 1 (Adaptive) *n* (%)	Cluster 2 (Maladaptive) *n* (%)	*p*	*V*
Sex	Male	19 (20.7)	20 (18.2)	0.658	0.031
	Female	73 (79.3)	90 (81.8)		
Education	Primary	13 (14.1)	23 (20.9)	0.407	0.120
	Lower Secondary	17 (18.5)	23 (20.9)		
	Higher Secondary	31 (33.7)	37 (33.6)		
	Higher Education	31 (33.7)	27 (24.5)		
Occupation	Unemployed	7 (7.6)	6 (5.5)	0.865	0.097
	Student	5 (5.4)	19 (9.1)		
	Housewife	43 (46.7)	54 (49.1)		
	Worker	16 (17.4)	18 (16.4)		
	Retired	5 (5.4)	7 (6.4)		
	Self-employed	16 (17.4)	15 (13.6)		
Anxiety Disorder		6 (6.5)	14 (12.7)	0.141	0.103
MDD		3 (3.3)	26 (23.6)	<0.001 *	0.289
BED		10 (10.9)	28 (25.5)	<0.01 *	0.186
FA		19 (20.7)	78 (70.9)	<0.001 *	0.501

* *p* < 0.05; *p* = significance level; *V* = Cramer’s V (effect size); MDD: Major Depressive Disorder; BED: Binge Eating Disorder; FA: Food Addiction.

## Data Availability

The original contributions presented in the study are included in the article, further inquiries can be directed to the corresponding author.

## References

[1] Courcoulas A.P., King W.C., Belle S.H., Berk P., Flum D.R., Garcia L., Gourash W., Horlick M., Mitchell J.E., Pomp A. (2018). Seven-year weight trajectories and health outcomes in the Longitudinal Assessment of Bariatric Surgery (LABS) study. JAMA Surg..

[2] Jackson V.M., Breen D.M., Fortin J.-P., Liou A., Kuzmiski J.B., Loomis A.K., Rives M.-L., Shah B., Carpino P.A. (2015). Latest approaches for the treatment of obesity. Expert Opin. Drug Discov..

[3] Field A.E., Camargo C.A., Ogino S. (2013). The merits of subtyping obesity: One size does not fit all. JAMA.

[4] Conceição E.M., Utzinger L.M., Pisetsky E.M. (2015). Eating disorders and problematic eating behaviours before and after bariatric surgery: Characterization, assessment and association with treatment outcomes. Eur. Eat. Disord. Rev..

[5] Wade K.H., Kramer M.S., Oken E., Timpson N.J., Skugarevsky O., Patel R., Bogdanovich N., Vilchuck K., Davey Smith G., Thompson J. (2017). Prospective associations between problematic eating attitudes in midchildhood and the future onset of adolescent obesity and high blood pressure. Am. J. Clin. Nutr..

[6] Gallant A., Lundgren J., Drapeau V. (2012). The night-eating syndrome and obesity. Obes. Rev..

[7] Burmeister J.M., Hinman N., Koball A., Hoffmann D.A., Carels R.A. (2013). Food addiction in adults seeking weight loss treatment. Implications for psychosocial health and weight loss. Appetite.

[8] Niego S.H., Kofman M.D., Weiss J.J., Geliebter A. (2007). Binge eating in the bariatric surgery population: A review of the literature. Int. J. Eat. Disord..

[9] Jiménez-Murcia S., Agüera Z., Paslakis G., Munguia L., Granero R., Sánchez-González J., Sánchez I., Riesco N., Gearhardt A.N., Dieguez C. (2019). Food Addiction in Eating Disorders and Obesity: Analysis of Clusters and Implications for Treatment. Nutrients.

[10] Ivezaj V., Wiedemann A.A., Grilo C.M. (2017). Food addiction and bariatric surgery: A systematic review of the literature. Obes. Rev..

[11] Guerrero Perez F., Sánchez-González J., Sánchez I., Jiménez-Murcia S., Granero R., Simó-Servat A., Ruiz A., Virgili N., López-Urdiales R., Montserrat-Gil de Bernabe M. (2018). Food addiction and preoperative weight loss achievement in patients seeking bariatric surgery. Eur. Eat. Disord. Rev..

[12] Cope E.C., Gould E. (2017). New evidence linking obesity and food addiction. Biol. Psychiatry.

[13] Michaud A., Vainik U., Garcia-Garcia I., Dagher A. (2017). Overlapping neural endophenotypes in addiction and obesity. Front. Endocrinol..

[14] Nightingale B.A., Cassin S.E. (2019). Disordered eating among individuals with excess weight: A review of recent research. Curr. Obes. Rep..

[15] Troisi A. (2022). Emergence of bariatric psychiatry as a new subspecialty. World J. Psychiatry.

[16] Williams G.A., Hawkins M.A., Duncan J., Rummell C.M., Perkins S., Crowther J.H. (2017). Maladaptive eating behavior assessment among bariatric surgery candidates: Evaluation of the Eating Disorder Diagnostic Scale. Surg. Obes. Relat. Dis..

[17] Rusch M.D., Andris D. (2007). Maladaptive eating patterns after weight-loss surgery. Nutr. Clin. Pract..

[18] Podina I.R., Fodor L.A., Cosmoiu A., Boian R. (2017). An evidence-based gamified mHealth intervention for overweight young adults with maladaptive eating habits: Study protocol for a randomized controlled trial. Trials.

[19] Chandrasekaran P., Weiskirchen R. (2024). The role of obesity in type 2 diabetes mellitus—An overview. Int. J. Mol. Sci..

[20] First M.B., Williams J.B., Karg R.S., Spitzer R.L. (2016). Structured Clinical Interview for DSM-5 Disorders: SCID-5-CV Clinician Version.

[21] Karlsson J., Persson L.O., Sjostrom L., Sullivan M. (2000). Psychometric properties and factor structure of the Three-Factor Eating Questionnaire (TFEQ) in obese men and women. Results from the Swedish Obese Subjects (SOS) study. Int. J. Obes. Relat. Metab. Disord. J. Int. Assoc. Study Obes..

[22] Stunkard A.J., Messick S. (1985). The three-factor eating questionnaire to measure dietary restraint, disinhibition and hunger. J. Psychosom. Res..

[23] Kıraç D., Kaspar E.Ç., Avcılar T., Çakır Ö.K., Ulucan K., Kurtel H., Deyneli O., Güney A.İ. (2015). Obeziteyle ilişkili beslenme alışkanlıklarının araştırılmasında yeni bir yöntem “Üç faktörlü beslenme anketi”. Clin. Exp. Health Sci..

[24] Allison K.C., Lundgren J.D., O’Reardon J.P., Martino N.S., Sarwer D.B., Wadden T.A., Crosby R.D., Engel S.G., Stunkard A.J. (2008). The Night Eating Questionnaire (NEQ): Psychometric properties of a measure of severity of the Night Eating Syndrome. Eat. Behav..

[25] Atasoy N., Saraçlı Ö., Konuk N., Ankaralı H., Güriz O., Akdemir A., Sevinçer G.M., Atik L. (2014). Gece Yeme Anketi-Türkçe Formunun psikiyatrik ayaktan hasta popülasyonunda geçerlilik ve güvenilirlik çalışması. Anadolu Psikiyatr. Derg..

[26] Gearhardt A.N., Corbin W.R., Brownell K.D. (2009). Preliminary validation of the Yale food addiction scale. Appetite.

[27] Sevinçer G.M., Konuk N., Bozkurt S., Saraçlı Ö., Coşkun H. (2015). Psychometric properties of the Turkish version of the Yale Food Addiction Scale among bariatric surgery patients. Anatol. J. Psychiatry.

[28] Derogatis L.R., Savitz K.L. (1999). The SCL-90-R, Brief Symptom Inventory, and Matching Clinical Rating Scales. The Use of Psychological Testing for Treatment Planning and Outcomes Assessment.

[29] Dağ I. (1991). Belirti Tarama Listesi (Scl-90-R)’nin Üniversite Öğrencileri için güvenirliği ve geçerliği. [Reliability and validity of the Symptom Check List (SCL-90-R) for university students.]. Türk Psikiyatr. Derg..

[30] Fraley C., Raftery A.E. (1998). How many clusters? Which clustering method? Answers via model-based cluster analysis. Comput. J..

[31] Rousseeuw P.J. (1987). Silhouettes: A graphical aid to the interpretation and validation of cluster analysis. J. Comput. Appl. Math..

[32] Nylund K.L., Asparouhov T., Muthén B.O. (2007). Deciding on the Number of Classes in Latent Class Analysis and Growth Mixture Modeling: A Monte Carlo Simulation Study. Struct. Equ. Model. Multidiscip. J..

[33] Field A.E., Inge T.H., Belle S.H., Johnson G.S., Wahed A.S., Pories W.J., Spaniolas K., Mitchell J.E., Pomp A., Dakin G.F. (2018). Association of Obesity Subtypes in the Longitudinal Assessment of Bariatric Surgery Study and 3-Year Postoperative Weight Change. Obesity.

[34] Carbone E.A., Rania M., D’Onofrio E., Quirino D., De Filippis R., Rotella L., Aloi M., Fiorentino V.T., Murphy R., Segura-Garcia C. (2024). The Greater the Number of Altered Eating Behaviors in Obesity, the More Severe the Psychopathology. Nutrients.

[35] Caroleo M., Primerano A., Rania M., Aloi M., Pugliese V., Magliocco F., Fazia G., Filippo A., Sinopoli F., Ricchio M. (2018). A real world study on the genetic, cognitive and psychopathological differences of obese patients clustered according to eating behaviours. Eur. Psychiatry.

[36] Leombruni P., Rocca G., Fassino S., Gastaldi F., Nicotra B., Siccardi S., Lavagnino L. (2014). An exploratory study to subtype obese binge eaters by personality traits. Psychother. Psychosom..

[37] Horsager C., Færk E., Lauritsen M.B., Østergaard S.D. (2021). Food addiction comorbid to mental disorders: A nationwide survey and register-based study. Int. J. Eat. Disord..

[38] Ogden L.G., Stroebele N., Wyatt H.R., Catenacci V.A., Peters J.C., Stuht J., Wing R.R., Hill J.O. (2012). Cluster analysis of the national weight control registry to identify distinct subgroups maintaining successful weight loss. Obesity.

[39] Green M., Strong M., Razak F., Subramanian S., Relton C., Bissell P. (2016). Who are the obese? A cluster analysis exploring subgroups of the obese. J. Public Health.

[40] Müller A., Claes L., Wilderjans T.F., De Zwaan M. (2014). Temperament subtypes in treatment seeking obese individuals: A latent profile analysis. Eur. Eat. Disord. Rev..

[41] Camacho-Barcia L., Lucas I., Miranda-Olivos R., Jimenez-Murcia S., Fernandez-Aranda F. (2023). Applying psycho-behavioural phenotyping in obesity characterization. Rev. Endocr. Metab. Disord..

[42] Tuschl R.J. (1990). From dietary restraint to binge eating: Some theoretical considerations. Appetite.

[43] Alqahtani R.M., Alhazmi A. (2025). Association between cognitive restraint, emotional eating, uncontrolled eating, and body mass index among health care professionals. Sci. Rep..

[44] Morin I., Bégin C., Maltais-Giguère J., Bédard A., Tchernof A., Lemieux S. (2018). Impact of Experimentally Induced Cognitive Dietary Restraint on Eating Behavior Traits, Appetite Sensations, and Markers of Stress during Energy Restriction in Overweight/Obese Women. J. Obes..

[45] Roberts C., Troop N., Connan F., Treasure J., Campbell I.C. (2007). The effects of stress on body weight: Biological and psychological predictors of change in BMI. Obesity.

[46] Aloi M., Liuzza M.T., Rania M., Carbone E.A., De Filippis R., Gearhardt A.N., Segura-Garcia C. (2024). Using latent class analysis to identify different clinical profiles according to food addiction symptoms in obesity with and without binge eating disorder. J. Behav. Addict..

[47] Meule A., Gearhardt A.N. (2014). Food addiction in the light of DSM-5. Nutrients.

[48] Hauck C., Cook B., Ellrott T. (2020). Food addiction, eating addiction and eating disorders. Proc. Nutr. Soc..

[49] Hutson P.H., Balodis I.M., Potenza M.N. (2018). Binge-eating disorder: Clinical and therapeutic advances. Pharmacol. Ther..

[50] Steward T., Menchon J.M., Jiménez-Murcia S., Soriano-Mas C., Fernandez-Aranda F. (2018). Neural network alterations across eating disorders: A narrative review of fMRI studies. Curr. Neuropharmacol..

[51] Xenaki N., Bacopoulou F., Kokkinos A., Nicolaides N.C., Chrousos G.P., Darviri C. (2018). Impact of a stress management program on weight loss, mental health and lifestyle in adults with obesity: A randomized controlled trial. J. Mol. Biochem..

[52] Willem C., Gandolphe M.C., Doba K., Roussel M., Verkindt H., Pattou F., Nandrino J.L. (2020). Eating in case of emotion dys-regulation, depression and anxiety: Different pathways to emotional eating in moderate and severe obesity. Clin. Obes..

[53] Martins L.B., Braga Tibães J.R., Sanches M., Jacka F., Berk M., Teixeira A.L. (2021). Nutrition-based interventions for mood disorders. Expert Rev. Neurother..

[54] Marx W., Moseley G., Berk M., Jacka F. (2017). Nutritional psychiatry: The present state of the evidence. Proc. Nutr. Soc..

[55] Sysko R., Hildebrandt T., Wilson G.T., Wilfley D.E., Agras W.S. (2010). Heterogeneity moderates treatment response among patients with binge eating disorder. J. Consult. Clin. Psychol..

[56] World Health Organization (2022). WHO European Regional Obesity Report 2022.

